# Supplementary material for: Impact of opinion dynamics on the public health damage inflicted by COVID-19 in the presence of societal heterogeneities
Source: Front Digit Health. 2023 Jun 8;5:1146178. doi: 10.3389/fdgth.2023.1146178 (PMC10285391; doi:10.3389/fdgth.2023.1146178)
Supplement: Supplementary file 1 [file Datasheet1.pdf]

# Supporting information I

## Equations of SARS-CoV-2 disease dynamics

### Developing the Clustered Epidemiological Differential Equations (CEDE) for COVID-19

Table 3 contains all the fundamental states in our model while Table 4 contains all SARS-CoV-2 transition parameters. Referring to the notations in Table 3, we index each term by the group the individual in the state belongs. More specifically, $S_c(t)$ is the fraction of individuals who are susceptible and cooperative at time $t$. $S_n(t), E_c(t), E_n(t), P_c(t), P_n(t), I_{cs}(t), I_{ns}(t), I_{ca}(t), I_{na}(t), H_c(t), H_n(t)$ may be defined similarly using Table 3 as a basis. Finally, $R_c(t), R_n(t)$ are respectively the fractions of cooperative and non-cooperative individuals who recover from COVID-19 at time $t$ while $D(t)$ denotes those that died due to COVID-19 at time $t$. Let $I(t)$ be the fraction of the *infectious* individuals at time $t$. That is, $I(t)$ is the fraction of individuals who can infect susceptible individuals through contact. Then,

$$I(t) = P_c(t) + P_n(t) + I_{cs}(t) + I_{ns}(t) + I_{ca}(t) + I_{na}(t)$$

Interaction between individuals corresponding to $S_c(t), S_n(t)$ and $I(t)$ spread the disease to the susceptibles and transform them to Exposed states $E_c(t), E_n(t)$ - refer to the yellow arrows in Fig 4. The natural progression of the disease changes $E_c(t)$ to either $P_c(t)$ or $I_{ca}(t)$ at a certain probability. Similarly, with a certain probability, $E_n(t)$ transitions to either $P_n(t)$ or $I_{na}(t)$. $P_c(t)$ would then transition to $I_{cs}(t)$, and then to either $H_c(t)$ or $R_c(t)$ or $D(t)$ while $P_n(t)$ would then transition to $I_{ns}(t)$ to either $H_n(t)$ or $R_n(t)$ or $D(t)$. In addition, $I_{ca}(t)$ and $I_{na}(t)$ respectively transition to $R_c(t)$ and $R_n(t)$. Finally, $H_c(t)$ and $H_n(t)$ transition to either $R_c(t)$ or $R_n(t)$ or $D(t)$ - refer to the blue arrows in Fig 4. The transitions are similar for the males and females, different age groups and races, healthy individuals, and individuals with underlying health conditions, but the fractions of those who recover in each populace are different.

Individuals in any group can get in physical proximity to one another at a certain rate. The rate is much higher if the individuals are of the same race and within the same age group. Meanwhile, only a fraction of these contacts spread the disease. We consider that the disease spread rate is the product of the rate at which individuals are in physical contact with one another and the fraction of such contacts between susceptibles and infectious individuals. The physical contact rates of the system are also known as the disease spread rates. The contact rate differs for the various age groups. We use $\phi$ to denote the physical rate of interaction

**Table 3. Fundamental compartments of the model**

| Compartment | Description |
|---|---|
| $S_c$ | Cooperative susceptible |
| $S_n$ | Non-cooperative susceptible |
| $E_c$ | Cooperative exposed individuals |
| $E_n$ | Non-cooperative exposed individuals |
| $P_c$ | Cooperative presymptomatic individuals |
| $P_n$ | Non-cooperative presymptomatic individuals |
| $I_{cs}$ | Cooperative symptomatic infected individuals |
| $I_{ns}$ | Non-cooperative symptomatic infected individuals |
| $I_{ca}$ | Cooperative asymptomatic infected individuals |
| $I_{na}$ | Non-cooperative asymptomatic infected individuals |
| $H_c$ | Cooperative hospitalized individuals |
| $H_n$ | Non-cooperative hospitalized individuals |
| $R_c$ | Cooperative recovered individuals |
| $R_n$ | Non-cooperative recovered individuals |
| $D$ | Dead |

between individuals of the same race and age group (youngest and middle age groups). Older people have smaller, more family-centric networks, and spend less time with others [16]. Thus, their rate of interaction is lower compared to the other groups. Therefore, we denote the disease spread rate between the oldest group of the same race with $m\phi$ where $m < 1$. Similarly, we denote the physical contact rate between the oldest group and the other groups with $\kappa\phi$ where $\kappa < 1$. In addition, we represent the physical contact rate between the youngest age group of different races with $a\phi$, middle age group of different races as $b\phi$, and oldest age group of different races with $c\phi$. We denote any other physical interaction rate with $x\phi$, where $a < 1$, $b < 1$, $c < 1$, $x < 1$. Similarly, we use $\alpha$ to represent the opinion spread rate. In this case, ideas are exchanged during interactions - which might lead to a change in opinion. Meanwhile, one may be converted by exposure to public awareness campaigns (which may have a stronger impact because the individual might be infected and experiences the symptoms acutely). We denote the rate of such opinion change as $\delta$.

Interaction between susceptible individuals i.e. $S_c(t)$, $S_n(t)$ and Infectious individuals i.e. $P_c(t)$, $P_n(t)$, $I_{cs}(t)$, $I_{ns}(t)$, $I_{ca}(t)$, $I_{na}(t)$ may spread the disease to the susceptibles and transform them to early incubators - refer to the yellow arrows in Fig 4. We assumed that those that are hospitalized, $H_c(t)$, $H_n(t)$, are isolated from the general public (quarantined) and hence cannot infect other susceptible nor can they convert the opinions of others. Natural progression of the disease change $E_c(t)$ to $P_c(t)$ to $I_{cs}(t)$ to either $H_c(t)$ or $R_c(t)$ or $D(t)$; also, $E_c(t)$ to $I_{ca}(t)$ to $R_c(t)$ - refer to the blue arrows in Fig 4. The transitions due to the natural progression of the disease are similar for cooperatives and non-cooperatives.

**Table 4. SARS-CoV-2 transition parameters**

| Parameters | Description of parameters |
|---|---|
| $\frac{1}{\lambda}$ | Expected time an exposed individual is in presymptomatic stage |
| $\frac{1}{\gamma}$ | Expected time an individual is in presymptomatic stage |
| $\frac{1}{\mu}$ | Expected time a symptomatic individual is in infection stage |
| $\frac{1}{\beta}$ | Expected time an asymptomatic individual is infectious |
| $\frac{1}{\mu}$ | Expected time a symptomatic individual is in late infection stage |
| $\frac{1}{\sigma}$ | Expected time an individual hospitalized |
| $p$ | Probability that an infected individual will be asymptomatic |
| $\eta$ | Probability that an individual at late infection stage is hospitalized |
| $\lambda$ | Probability that an individual at late infection stage dies |

We model the evolution of the states through a set of meta-population epidemiological differential equations. Each differential equation captures the evolution of a particular variable. Thus, the solution of the system of differential equations provides the fraction of individuals in different states at given times, that is, the spatio-temporal distribution of the disease and opinion spread. The terms in the differential equations are either quadratic or linear. The quadratic ones represent the transitions brought on by interactions between two individuals, specifically physical proximity, and exchange of ideas (refer to the yellow arrows and the black arrows in Fig 4) and the linear ones represent the transitions that happen otherwise, specifically natural progression of the disease, conversion through reading, media, etc. (refer to the blue and black arrows in Fig 4). Note that interactions always involve two individuals, hence interactional transitions are represented by quadratic terms; in contrast, the non-interactional transitions involve only one individual and are therefore represented by linear terms.

Let $I(t)$ be the fraction of the infectious individuals at time $t$ and $X(t)$ be the fraction of individuals who are cooperative at time $t$. The resulting clustered epidemiological differential equations (CEDE) are given in equations (1) - (15).

$$\dot{S}_c(t) = -\sum S_c(t)\phi I(t) + \sum S_n(t)\alpha X(t) + \delta S_n(t) \quad (1)$$

$$\dot{S}_n(t) = -\sum S_n(t)\phi I(t) - \sum S_n(t)\alpha X(t) - \delta S_n(t) \quad (2)$$

$$\dot{E}_c(t) = \sum S_c(t)\phi I(t) + \sum E_n(t)\alpha X(t) + \delta E_n(t) - \lambda E_c(t) \quad (3)$$

$$\dot{E}_n(t) = \sum S_n(t)\phi I(t) - \sum E_n(t)\alpha X(t) - \delta E_n(t) - \lambda E_n(t) \quad (4)$$

$$\dot{P}_c(t) = (1-p)\lambda E_c(t) - \gamma P_c(t) + \delta P_n(t) + \sum P_n(t)\alpha X(t) \quad (5)$$

$$\dot{P}_n(t) = (1-p)\lambda E_n(t) - \gamma P_n(t) - \delta P_n(t) - \sum P_n(t)\alpha X(t) \quad (6)$$

$$\dot{I}_{cs}(t) = \gamma P_c(t) - \mu I_{cs_1}(t) + \delta I_{ns}(t) + \sum I_{ns}(t)\alpha X(t) \quad (7)$$

$$\dot{I}_{ca}(t) = p\lambda E_c(t) - \beta I_{ca}(t) + \delta I_{na}(t) + \sum I_{na}(t)\alpha X(t) \quad (8)$$

$$\dot{I}_{ns}(t) = \gamma P_n(t) - \mu I_{ns}(t) - \delta I_{ns}(t) - \sum I_{ns}(t)\alpha X(t) \quad (9)$$

$$\dot{I}_{na}(t) = p\lambda E_n(t) - \beta I_{na}(t) - \delta I_{na}(t) - \sum I_{na}(t)\alpha X(t) \quad (10)$$

$$\dot{H}_c(t) = \eta\mu I_{cs}(t) - \sigma H_c(t) + \delta H_n(t) \quad (11)$$

$$\dot{H}_n(t) = \eta\mu I_{ns}(t) - \sigma H_n(t) - \delta H_n(t) \quad (12)$$

$$\dot{R}_c(t) = (1-\eta)\mu I_{cs}(t) + (1-\lambda)\sigma H_c(t) + \mu I_{ca}(t) + \delta R_n(t) + \sum R_n(t)\alpha X(t) \quad (13)$$

$$\dot{R}_n(t) = (1-\eta)\mu I_{ns}(t) + (1-\lambda)\sigma H_n(t) + \mu I_{na}(t) - \delta R_n(t) - \sum R_n(t)\alpha X(t) \quad (14)$$

$$\dot{D}(t) = \sigma\theta H_c(t) + \sigma\theta H_n(t) \quad (15)$$

The terms in green color are quadratic terms that represent the spread of the SARS-CoV-2 to the susceptibles due to physical interaction with the infectious individuals and the subsequent transformation of the susceptibles to the exposed states - refer to the yellow arrows in Fig 4. The terms in orange represent the transformation of non-cooperative individuals to cooperative ones - refer to the black arrows in Fig 4. The quadratic terms represent those conversions due to the exchange of opinions during interaction whereas the linear terms represent changes in opinion due to public awareness campaigns. The terms in red represent the natural progression of the disease - refer to the blue arrows in Fig 4. The differential equations for the evolution of COVID-19 in cooperatives are identical to those of the non-cooperatives, as can be seen in equations 1 to 15.

Note that (1) is similar to (2) as transitions are similar for the cooperative and non-cooperative individuals.

The same observation may be made for $((3),(4))$, $((5),(6))$, $((7),(8))$, $((9),(10))$, $((11),(12))$, $((13),(14))$. So, we only explain (1), (3), (5), (7), (9), (11), (13), and (15).

The first terms in (1), (3), (the terms in green color) are quadratic terms that represent the spread of the disease to the susceptibles due to interaction with the infectious individuals and the subsequent transformation of the susceptibles to early incubators - refer to the yellow arrows in Fig 4. The rate of conversion of susceptibles in a group to the exposed stage is proportional to the number of physical contacts per unit time between the susceptibles with infectious individuals since each such contact spreads the disease to the susceptible with a certain probability. The proportionality constant here is the probability that such a contact spreads the disease or equivalently the fraction of such contacts that spread the disease. The number of such physical contacts per unit time again is proportional to the number of pairs of susceptibles in the group in question and infectious in the same and other groups. Here the proportionality constant is the reciprocal of the expected time between successive physical contacts between individuals in a given such pair. The expected time will presumably be lower, and therefore the proportionality constant higher, if both individuals are in the same group than if they are in different groups since connections between individuals of the same age, race, and gender are usually more frequent [16–18]. The number of pairs of individuals one of which is susceptible in one group and another an infectious individual in the same or other groups is the product of the number of susceptibles in one group and infectious in the same or another group. These products lead to the quadratic terms. The overall proportionality constants are the products of the two proportionality constants mentioned above, and give us $\phi$, the disease spread rates, which have been summarized in Table 8. Since the spread of the disease reduces the number of susceptibles and increases the number of early incubators, the first term in (1) has a positive sign and that in (3) has a negative sign.

The second terms in (1) through (14) (the terms in orange) are quadratic terms that represent the transformation of non-cooperative individuals to cooperative ones through the exchange of opinions with cooperative individuals - refer to the black arrows in Fig 4. The rate of transformation for non-cooperatives of a certain type (the type is specified by the stage of disease, cooperativity, health status, gender, race, and age) is proportional to the number of opinion exchanges per unit time between the non-cooperatives and cooperatives; the proportionality constant is the value of the above fraction. The number of such exchanges per unit time again is proportional to the number of pairs of non-cooperatives of the type in question and cooperatives in the same and other groups. The number of pairs of individuals one of which is a non-cooperative of a certain type and another a cooperative in the same or another group is the product of the

number of non-cooperatives of the type and cooperatives in the same or other groups. These products lead to the quadratic terms. The overall proportionality constant is the product of the two proportionality constants above, and provides us the opinion spread rates, $\alpha$. The third terms in (1) through (14) (the terms in orange) are linear terms that represent the transformation of non-cooperative individuals to cooperative ones through exposure to public awareness campaigns (which may have a stronger impact because the individual might be infected and thus experiences the symptoms acutely). We denote the rate of such opinion change as $\delta$. Since the change of opinion increases (respectively decreases) the cooperatives (respectively non-cooperatives), the second terms in the equations for the cooperatives (e.g., (1)) are positive and the second terms for the non-cooperatives (e.g., (2)) are negative.

All other terms in (3), (5), (7), (9), (11), (13), and (14) (the terms in red) represent the natural progression of the disease - refer to the yellow arrows in Fig 4. For instance, the second term in (5) and the first term in (7) represent the natural progression from the presymptomatic to the symptomatic infectious state. The first term in (13) represents the recovery of cooperative individuals from the symptomatic infected stage. Finally, the solution of the CEDE provides the spatio-temporal distribution for the spread of the disease, namely the fraction of individuals who (1) are dead in any group at time $t$ ($D(t)$); (2) have recovered in any group at time $t$ ($R(t)$); (3) are susceptible and are in any group at time $t$ ($(Sc + S_h)(t)$); (4) are infected in any group at time $t$ ($(P_{ci} + P_{hi} + C_{ci} + C_{hi} + E_{ci} + E_{hi})(t)$); (5) are contagious and are in cluster $i$ at time $t$ ($(P_c + P_n + I_{cs} + I_{ns} + I_{ca} + I_{na})(t)$).

Meanwhile, equations (1) - (15) represent the basic compartments in our model. We now generalize the states in our model. Recall that an individual is characterized by his cooperativity, health conditions, gender, ancestry, age, stage of the disease, as well as symptomatic or asymptomatic manifestation. Thus, we use the suffixes $c$ to denote cooperativity and $n$ to denote non-cooperativity. Similarly, $h$ denotes being healthy (immunocompetent), $d$ denotes immunodeficient (not healthy, immunocompromised), $m$ represents male, while $f$ represents female. In addition, we use the subscripts $x$, $y$, and $z$ respectively to represent individuals of African American, Hispanic, and White-American ancestry. Furthermore, we classify the age of an individual into three groups with subscript 1 denoting $0 - 24$ years, 2 denoting $25 - 49$ years, and 3 denoting 50 years and above. Finally, we use the suffixes $s$ and $a$ to respectively denote symptomatic and asymptomatic individuals. Therefore, the state $S_{chmx1}$ denotes cooperative, healthy, male susceptible African American in the $0 - 24$ years age group. Similarly, $E_{ndfx3}$ denotes non-cooperative, not healthy (immunocompromised), exposed female Hispanic/Latino who is 50 years and above, $I_{chmzs2}$ denotes cooperative, healthy, infected

symptomatic male White-American in the $25-49$ years age group while $I_{ndfza1}$ denotes non-cooperative, not healthy (immunocompromised), infected asymptomatic female African American in the $0-24$ years age group.

# Supporting information II

## Parameter estimation

Table 5 shows the demographic characteristics of Pennsylvania with respect to age and race [23]. Note that few percentages of the population are neither African Americans, Hispanics/Latinos, nor White-American. Thus, we distributed such population according to the percentage of those 3 races we considered. For instance, for the age group 25 - 49 years, there are 4.4% African Americans, 1.3% Hispanics, 24.0% White-American, and 2.0% other races. We distributed the 2.0% population thus: African Americans = 4.4 + (4.4 × 2)/29.7 = 4.7%. Similarly, Hispanics = 1.3 + (1.3 × 2)/29.7 = 1.4%, while White-American = 24.0 + (24 × 2)/29.7 = 25.6%. In addition, the gender distribution in Pennsylvania is 51.1% female and 48.9% male [35]. The total number of people we considered in our model is 10 million whereas our choice for the initial number of infected individuals is 10000.

**Table 5. Population distribution by race and age.**

| Age (years) | African/Black (%) | Hispanic/Latino (%) | White (%) | Total (%) |
|---|---|---|---|---|
| 0 - 24 | 5.9 | 2.3 | 22.6 | 30.8 |
| 25 – 49 | 4.7 | 1.4 | 25.6 | 31.7 |
| 50+ | 3.2 | 0.7 | 33.6 | 37.5 |
| Total | 13.8 | 4.4 | 81.8 | 100 |

Comorbidity refers to the existence of more than one disease or condition in the same person at the same time. The Centers for Disease Control and Prevention (CDC) and the PA Department of Health (PaDOH) have highlighted concern for individuals of all ages who suffer from underlining medical conditions such as obesity, as well as those who are immunocompromised due to conditions like cancer treatment and being HIV/AIDS positive [12]. Furthermore, according to [36], about 40% of the US population are immunocompromised. Body Mass Index (BMI) is a person's weight in kilograms divided by the square of height in meters. BMI over 30 is considered obese [22]. The average obesity rate among African American/Black, Hispanic/Latino, and White in Pennsylvania are 41.8%, 32.9%, and 31.3% respectively [37]. In this paper, we refer to people without any comorbidities as healthy. We obtain the fraction of people with underlying medical conditions by

taking the average of immunocompromised and obese individuals. Thus, comorbidity for Africa America is (0.2 + 0.418)/2 = 0.3090; Hispanic = (0.2 + 0.329)/2 = 0.2645; and White = (0.2 + 0.313)/2 = 0.2565.

According to the Centers for Disease Control and Prevention (CDC), the current best estimate of the basic reproduction number, $R_0$ for COVID-19 is 2.5 [11]. Recall that $R_0$ is the average number of secondary infections caused by a single typical infected individual among a completely susceptible population. If $R_0 > 1$, epidemic takes off. On the other hand, if $R_0 < 1$, no major epidemic occurs. In addition, following the procedure outlined in (Chapter 6, [38]), we obtain the expression for $R_0$ for our model as shown in equation 16.

$$R_0 = \frac{p\phi N}{\beta} + \frac{(1-p)\phi N}{\gamma} + \frac{\eta(1-p)\phi N}{\sigma} + \frac{(1-\eta)(1-p)\phi N}{\mu} \tag{16}$$

Thus,

$$\begin{aligned} 2.5 &= \frac{0.3 \times \phi \times 10^7}{1/10} + \frac{(1-0.3) \times \phi \times 10^7}{1/2} + \frac{(0.12 \times (1-0.3) \times \phi \times 10^7)}{1/5} \\ &\quad + \frac{(1-0.12) \times (1-0.3) \times \phi \times 10^7}{1/9} \\ 2.5 &= 3 \times 10^7\phi + 1.4 \times 10^7\phi + 0.42 \times 10^7\phi + 5.544 \times 10^7\phi \\ \phi &= 2.4122 \times 10^{-8} \text{ per person per day} \end{aligned}$$

Furthermore, 30% of infected individuals are asymptomatic [11]. The transmission of SARS-CoV-2 from an infected person to a secondary patient before the source patient developed symptoms is known as presymptomatic transmission [39]. The presymptomatic stage lasts for 2 days on average [40], [39]. The mean time from exposure to symptom onset is 6 days [11], i.e., $1/\lambda + 1/\gamma = 6$ days. But $1/\gamma = 2$ days on average [40]. Therefore, $1/\lambda = 4$ days, and $1/\gamma = 2$ days. The median number of days from symptom onset to hospitalization is 5 days [11]. That is expected time $1/\phi + 1/\beta = 5$ days. Therefore, $1/\beta = 2$ days. The median number of days from symptom onset to death is 15 days [11]. Therefore, $1/\sigma =$ 15 - $1/\mu - 1/\beta =$ 15 – 3 – 3 = 9 days. Thus, $1/\sigma = 9$ days. Furthermore, the median duration of hospital stays among survivors = 9.3 days [41]. Similarly, according to CDC, the persons who never develop symptoms, isolation, and other precautions can be discontinued 10 days after the date of their first positive RT-PCR test for SARS-CoV-2 RNA. Therefore, $1/\beta = 10$ days [42, 43]. The percent that dies among those hospitalized is 0.7% (0 – 17 years old), 2.1% (18 – 49 years old), 7.9% (50 – 64 years old), 18.8% ($\geq$ 65 years old) [11]. Therefore, fatality

**Table 6. Parameter estimation**

| Parameter | Description | Value | Ref. |
|---|---|---|---|
| $p$ | Probability that an infected person is asymptomatic | 0.3 | [11] |
| $\eta$ | Probability that an infected person is hospitalized | Table 7 | [45] |
| $\lambda$ | COVID-19 fatality rate | see p. 40 | [11] |
| $\frac{1}{\lambda}$ | Expected time an individual is in exposed stage | 4 days | p. 40 |
| $\frac{1}{\gamma}$ | Expected time an individual is pre-symptomatic | 2 days | [39, 40] |
| $\frac{1}{\mu}$ | Expected time an individual is infected before hospitalization | 5 days | [11] |
| $\frac{1}{\sigma}$ | Expected time for a hospitalized individual to recovers | 9 days | [41], p. 40 |
| $\frac{1}{\mu}$ | Expected time for an infected asymptomatic individual to recover | 10 days | [42, 43] |
| $R_0$ | Basic Reproduction number | 2.5 | [11] |
| $\phi$ | Disease spread rate | $2.4122 \times 10^{-8}$ | p. 40 |

rates among hospitalized patients are: $(0.7 + 2.1)/2 = 1.4\%$ for $0 - 24$ years, 2.1% for $25 - 49$ years, and $(7.9 + 18.8)/2 = 13.35\%$ for 51+ years.

Similar to [24], we define the fractions of individuals who fully comply with COVID-19 prevention measures at the initial time as the *initial cooperativity*. According to [36], approximately 40% of the populace is not cooperative. Thus, our default choice for the initial cooperativity is 0.6, but we also consider other values of initial cooperativity. We assume that interaction between a pair of non-cooperatives is twice likely to result in infection. In addition, we assume that the disease spread rate between a pair of individuals such that only one is non-cooperative equals that when both are non-cooperatives. COVID-19 vaccines have been shown to be effective and help to reduce hospitalizations, intensive care unit admissions, and deaths [7, 8]. However, the secondary attack rate among household contacts exposed to fully vaccinated index cases was similar to household contacts exposed to unvaccinated index cases [44]. Meanwhile, vaccination against COVID-19 is now seamless and vaccines are readily available to receive across the U.S. Thus, we also considered cases in which non-cooperatives are assumed to be twice likely to be hospitalized compared with cooperatives. The parameters we use in this research alongside their estimated values are outlined in Table 6.

Next, we use data from [45] to estimate the probability of hospitalization for African Americans, Hispanics/Latinos, and White Americans for the various age groups. We assumed that each individual averages 1 –

4 minutes of exercise per day. We also assumed that a healthy individual weighs 147 lbs on average while an obese individual weighs 203 lbs or more.

**Table 7. The average probability of hospitalization** [45].

| Age (years) | Sex | Health condition | Probability of hospitalization | | |
|---|---|---|---|---|---|
| | | | Africa Americans | Hispanics | Whites |
| 0 - 24s | M | Healthy | 0.03 | 0.03 | 0.03 |
| 0 - 24s | F | Healthy | 0.03 | 0.03 | 0.03 |
| 0 - 24s | M | Comorbid | 0.19 | 0.14 | 0.12 |
| 0 - 24s | F | Comorbid | 0.16 | 0.11 | 0.09 |
| 25 - 49s | M | Healthy | 0.05 | 0.03 | 0.03 |
| 25 - 49s | F | Healthy | 0.04 | 0.03 | 0.03 |
| 25 - 49s | M | Comorbid | 0.25 | 0.18 | 0.16 |
| 25 - 49s | F | Comorbid | 0.21 | 0.15 | 0.13 |
| 50+ | M | Healthy | 0.15 | 0.10 | 0.09 |
| 50+ | F | Healthy | 0.12 | 0.08 | 0.08 |
| 50+ | M | Comorbid | 0.50 | 0.40 | 0.36 |
| 50+ | F | Comorbid | 0.44 | 0.35 | 0.31 |

We use the subscripts $x$, $y$, and $z$ to respectively represent people of African American, Hispanic, and White American ancestry. We also classify the age of an individual into three groups with subscript 1 denoting $0-24$ years, 2 denoting $25-49$ years, and 3 denoting 50 years and above. We use $\phi$ to denote the physical rate of interaction between individuals of the same race and age group (youngest and middle age groups). Older people have smaller, more family-centric networks, and spend less time with others [16]. Thus, their rate of interaction is lower compared to the other groups. Therefore, we denote the disease spread rate between the oldest group of the same race with $m\phi$ where $m < 1$. Similarly, we denote the physical contact rate between the oldest group and the other groups with $\kappa\phi$ where $\kappa < 1$. In addition, we represent the physical contact rate between the youngest age group of different races with $a\phi$, middle age group of different races as $b\phi$, and oldest age group of different races with $c\phi$. We denote any other physical interaction rate with $x\phi$, where $a < 1$, $b < 1$, $c < 1$, and $x < 1$. As shown above, $\phi = 2.4122 \times 10^{-8}$ per person per day, $a = 0.9$,

$b = 0.8$, $c = 0.2$, $k = 0.6$, $x = 0.3$, and $m = 0.4$. Similarly, we use $\alpha$ to represent the virtual interaction rate also known as the opinion spread rate. We assumed that $\alpha = 10^{-9}$ and $\delta = 2 \times 10^{-9}$ (default values).

**Table 8. Interaction rates.**

| | X1 | X2 | X3 | Y1 | Y2 | Y3 | Z1 | Z2 | Z3 |
|---|---|---|---|---|---|---|---|---|---|
| X1 | $\phi$ | $\phi$ | $k\phi$ | $a\phi$ | $x\phi$ | $x\phi$ | $a\phi$ | $x\phi$ | $x\phi$ |
| X2 | $\phi$ | $\phi$ | $k\phi$ | $x\phi$ | $b\phi$ | $x\phi$ | $x\phi$ | $b\phi$ | $x\phi$ |
| X3 | $k\phi$ | $k\phi$ | $m\phi$ | $x\phi$ | $x\phi$ | $c\phi$ | $x\phi$ | $x\phi$ | $c\phi$ |
| Y1 | $a\phi$ | $x\phi$ | $x\phi$ | $\phi$ | $\phi$ | $k\phi$ | $a\phi$ | $x\phi$ | $x\phi$ |
| Y2 | $x\phi$ | $b\phi$ | $x\phi$ | $\phi$ | $\phi$ | $k\phi$ | $x\phi$ | $b\phi$ | $x\phi$ |
| Y3 | $x\phi$ | $x\phi$ | $c\phi$ | $k\phi$ | $k\phi$ | $m\phi$ | $x\phi$ | $x\phi$ | $c\phi$ |
| Z1 | $a\phi$ | $x\phi$ | $x\phi$ | $a\phi$ | $x\phi$ | $x\phi$ | $\phi$ | $\phi$ | $k\phi$ |
| Z2 | $x\phi$ | $b\phi$ | $x\phi$ | $x\phi$ | $b\phi$ | $x\phi$ | $\phi$ | $\phi$ | $k\phi$ |
| Z3 | $x\phi$ | $x\phi$ | $c\phi$ | $x\phi$ | $x\phi$ | $c\phi$ | $k\phi$ | $k\phi$ | $m\phi$ |

# Supporting Information III

## Supplementary figures

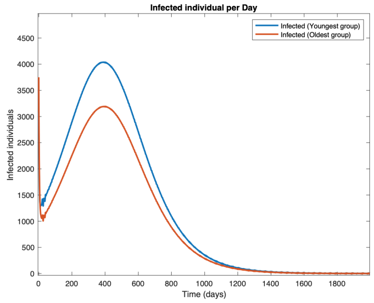


**(a)** Infection per day

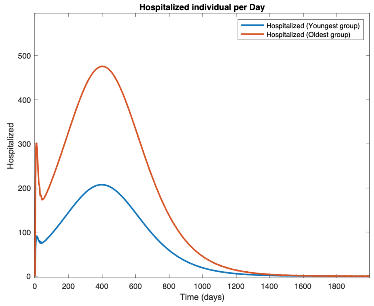


**(b)** Hospitalization per day

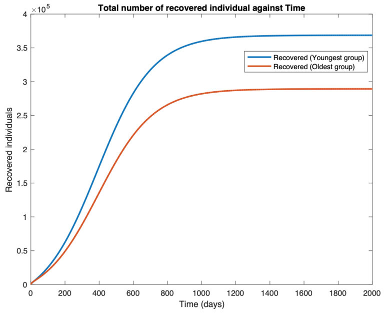


**(c)** Number of recovered individuals

**Fig 16. Scenario 1.** Plots in the time domain for sanity check.

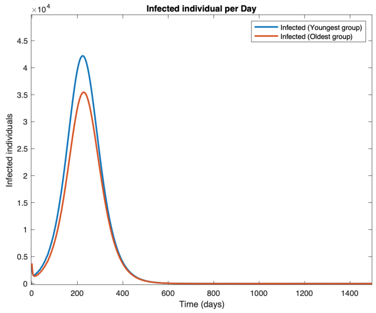


**(a)** Infection per day

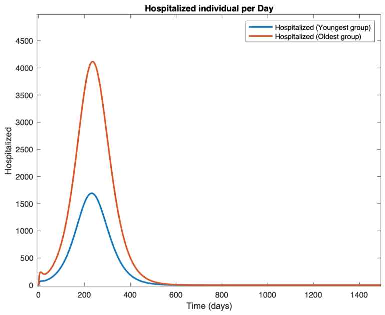


**(b)** Hospitalization per day

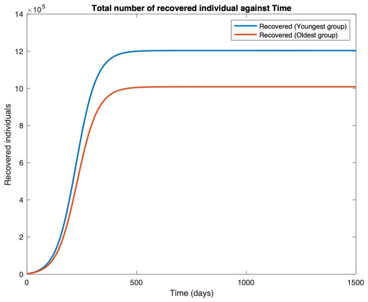


**(c)** Number of recovered individuals

**Fig 17. Scenario 2.** Plots in the time domain for sanity check.

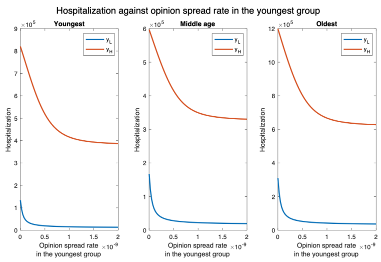


**(a)** Opinion spread rate varied for the youngest group

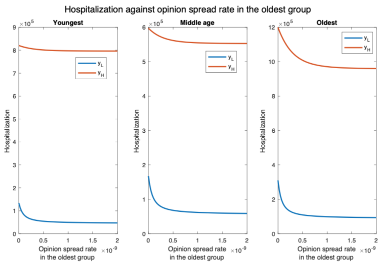


**(b)** Opinion spread rate varied for the oldest group

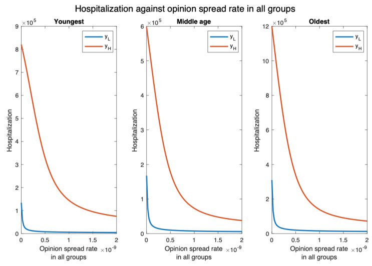


**(c)** Opinion spread rate varied for all the groups

**Fig 18.** Hospitalization count for various interaction rates of the youngest group. $y_L$ denotes the default interaction rates of the youngest group while $y_H$ represent when the youngest group interaction rates are tripled (other groups interaction rates remain the same).

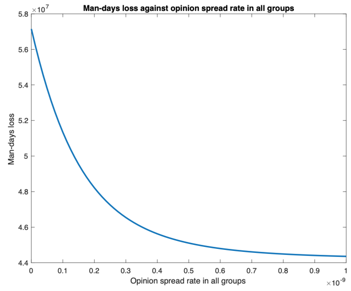


**(a)** Opinion spread rate varied for all the groups

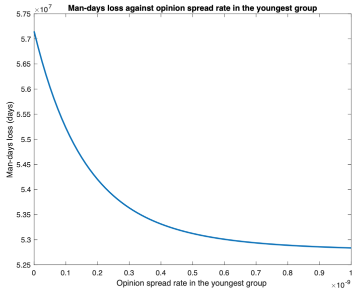


**(b)** Opinion spread rate varied for the youngest group

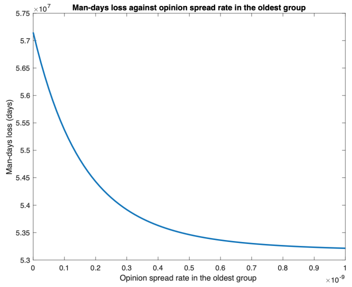


**(c)** Opinion spread rate varied for the oldest group

**Fig 19.** Impact of long COVID when vaccines are administered.

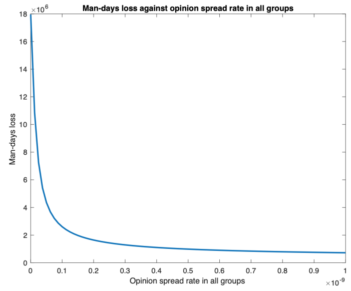


**(a)** Opinion spread rate varied for all the groups

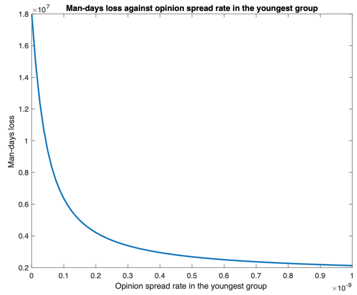


**(b)** Opinion spread rate varied for the youngest group

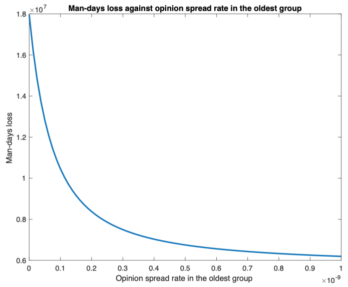


**(c)** Opinion spread rate varied for the oldest group

**Fig 20.** Impact of long COVID for the joint implementation of vaccines and behavioral practices.

# References

7. Centers for Disease Control and Prevention (CDC). Summary of guidance for minimizing the impact of COVID-19 on individual persons, communities, and Health Care Systems - United States. https://www.cdc.gov/mmwr/volumes/71/wr/mm7133e1.htm. Aug 2022. Accessed: November 1, 2022.

8. Thompson, M. G., Stenehjem, E., Grannis, S., Ball, S. W., Naleway, A. L., Ong, T. C., DeSilva, M. B., Natarajan, K., Bozio, C. H., Lewis, N., et al. Effectiveness of covid-19 vaccines in ambulatory and inpatient care settings. *New England Journal of Medicine 385*, 15 (2021), 1355–1371.

11. Centers for Disease Control and Prevention. COVID-19 Pandemic Planning Scenarios. https://www.cdc.gov/coronavirus/2019-ncov/hcp/planning-scenarios.html. Accessed: December 10, 2020.

12. Chandler RF. COVID19 and The Commonwealth: Vulnerable Populations in Pennsylvania: Health Risks & Factors. Pennsylvania Population Network, Pennsylvania State University, University Park, PA. Issue 05, Apr 2020.

16. Marcum, C. S. Age differences in daily social activities. *Research on aging 35*, 5 (2013), 612–640.

17. Vaidyanathan, R. Why Don't Black and White Americans Live Together? *British Broadcasting Corporation, January 8* (2016).

18. Dunsmuir, L. Many Americans have no friends of another race: Poll. *Reuters, August 8* (2013).

22. Centers for Disease Control and Prevention (CDC). Defining Adult Overweight & Obesity. https://www.cdc.gov/obesity/basics/adult-defining.html. Accessed: February 6, 2021.

23. Statistical Atlas. The Demographic Statistical Atlas of the United States.

https:statisticalatlas.com/statecom/state/Pennsylvania/Age-and-Sex. Accessed: July 20, 2021.

24. Ali, R. N., Rubin, H., and Sarkar, S. Countering the potential re-emergence of a deadly infectious disease—Information warfare, identifying strategic threats, launching countermeasures. *Plos one 16*, 8 (2021), e0256014.

35. World Population Review. Pennsylvania Gender and Religion Statistics. https://worldpopulationreview.com/states/pennsylvania-population. Accessed: August 28, 2022.

36. Moore, R. C., Lee, A. Y., Hancock, J. T., Halley, M. C., and Linos, E. Age-related differences in experiences with social distancing at the onset of the COVID-19 pandemic: A computational and content analytic investigation of natural language from a social media survey. *JMIR human factors 8*, 2 (2021), e26043.

37. Allen, T. About a third of Pennsylvanians self-reported obesity in 2020. https://www.axios.com/local/philadelphia/2021/10/12/pennsylvania-obesity-rate-cdc, Oct 2021.

38. Smith, G. *Back of the Envelope Modelling of Infectious Disease Transmission Dynamics for Veterinary Students*. Cambridge Scholars Publishing, 2019.

39. Morbidity and Mortality Weekly Report (MMWR), Centers for Disease Control and Prevention (CDC). Presymptomatic transmission of SARS-COV-2 - singapore, January 23 – March 16, 2020. 2020 Dec. https://www.cdc.gov/mmwr/volumes/69/wr/mm6914e1.htm#:~:text=Presymptomatic%20transmission%20was%20defined%20as,exposed%20to%20anyone%20else%20with, Apr 2020. Accessed: July 20, 2021.

40. World Health Organization (WHO). Coronavirus disease (COVID-19): How is it transmitted? https://www.who.int/news-room/questions-and-answers/item/ coronavirus-disease-covid-19-how-is-it-transmitted. Accessed: July 20, 2021.

41. Lewnard, J. A., Liu, V. X., Jackson, M. L., Schmidt, M. A., Jewell, B. L., Flores, J. P., Jentz, C., Northrup, G. R., Mahmud, A., Reingold, A. L., et al. Incidence, clinical outcomes, and transmission dynamics of severe coronavirus disease 2019 in California and Washington: prospective cohort study. *bmj 369* (2020).

42. Centers for Disease Control and Prevention (CDC). Ending Isolation and Precautions for People with COVID-19: Interim Guidance. 2020 Dec. https://www.cdc.gov/coronavirus/2019-ncov/hcp/duration-isolation.html. Accessed: July 20, 2021.

43. World Health Organization (WHO). Criteria for releasing COVID-19 patients from isolation. https://www.who.int/news-room/commentaries/detail/ criteria-for-releasing-covid-19-patients-from-isolation. Accessed: August 28, 2022.

44. Singanayagam, A., Hakki, S., Dunning, J., Madon, K. J., Crone, M. A., Koycheva, A., Derqui-Fernandez, N., Barnett, J. L., Whitfield, M. G., Varro, R., et al. Community transmission and viral load kinetics of the SARS-CoV-2 delta (B. 1.617. 2) variant in vaccinated and unvaccinated individuals in the UK: a prospective, longitudinal, cohort study. *The Lancet Infectious Diseases 22*, 2 (2022), 183–195.

45. 23andMe, Inc. COVID-19 Information Center. https://you.23andme.com/covid19/. Accessed: July 20, 2021.
